# Understanding Female Students’ Needs to Develop Health Promoting School Programme: An Exploratory Qualitative Analysis

**DOI:** 10.21315/mjms2018.25.2.9

**Published:** 2018-04-27

**Authors:** Fatemeh Rakhshani, Ali-Reza Ansari Moghaddam, Fariba Shahraki-Sanavi, Mahdi Mohammadi, Saeed Fakhrerahimi

**Affiliations:** 1 Safety Promotion and Injury Prevention Research Center (SPIPRC), Public Health Department, Shahid Beheshti University of Medical Sciences, Tehran, 1983963113 Iran (the Islamic Republic of); 2 Health Promotion Research Center, Public Health Department, Doctor Hesabi Square, Zahedan University of Medical Sciences, Zahedan, Sistan and Baluchestan, 9817667993 Iran (the Islamic Republic of); 3 Parent-Teacher Association Office, Education and Training Sistan and Baluchestan Province, Zahedan, 9816913415 Iran (the Islamic Republic of)

**Keywords:** promoting school programme, needs, female, Iran, exploratory qualitative study

## Abstract

**Background:**

This study was carried out on Iranian female adolescents to understand health needs for the purpose of designing health promoting intervention in schools.

**Methods:**

In this exploratory qualitative study, two focus group discussion (15 teachers) and 30 individual in-depth interviews were conducted among female adolescents in the eighth grade in Zahedan, Iran. Qualitative content analysis was used for data evaluation.

**Results:**

The views of students and teachers demonstrated nine of needs including: informing students about the schools’ health project aims, education and training all dimensions of health with an emphasis on mental health, use of experts in various fields for education from other organisations, employing capable and trusted counselors in schools, utilisation of a variety of teaching methods, activating reward systems for encouraging students’ participation in group activities, teaching communication and the ability to establish good relationships with parents and strategies for resolving family conflict, teaching parents and students high-risk behaviours and strategies for handling them as well as reforming wrong attitudes and indigenous sub-culture.

**Conclusion:**

This study found the different needs of Iranian female students compared to other cultures about a health promoting school programme. Therefore, their contribution can provide an insight for formulating policies and intervention in schools.

## Introduction

Having a connection and a sense of belonging to the school environment protects children and adolescents from many of the threats to their health, education and social welfare. It has also been widely recognised as a factor involved in promoting their emotional and psychological health (1, 2). Furthermore, this special relationship protects students from committing high-risk behaviours such as drug abuse, violence, sexual relationships and the tendency to drink or abuse alcohol (3–7).

There had been some evidence that adolescents’ perception of the sense of belonging to the school’s social environment has a significant positive relationship with students’ academic achievement (1, 8, 9), and the development into adulthood as well (10). In contrast, adolescents who have been marginalised at the school or expelled are more likely to be involved in high-risk and abnormal social behaviours (11).

Accordingly, the World Health Organization (WHO) has begun to establish health-promoting schools throughout the world (12). In general, health-promoting schools provide students with a specific education (health education and physical activity), healthcare (health, psychological and counseling services), a good physical environment and socio-cultural systems (13).

Indeed, the approaches of these schools involve preparation of the school’s position as a health-promoting environment. In this model, the school is considered as a whole entity which has its own curriculum, teaching and learning, policies, organisation and procedures, physical and social environment, as well as links to families, social services and organisations (14–16). Students are said to be at the heart of this model, because they are surrounded by the school environment as well as structural and organisational skills (17).

In accordance with the WHO, creating a healthy school implies adopting a new way of thinking about health and the role of schools in this matter (17). The framework defined by the WHO regards healthy schools as organised policies, methods, infrastructures and activities that protect and promote the health and well-being of the students, teachers, directors, authorities and the society as a whole (18). In addition, numerous studies have explored the effectiveness of these interventions on all the dimensions of health among the students, as well as the control and prevention programmes aimed at smoking, alcohol and drug use, weight fluctuations, sexually transmitted diseases, mental health, etc. (19–23).

Nevertheless, some studies demonstrated the various challenges that might be encountered by health-promoting schools (15, 24). For example, one of the main challenges of implementing this project in the US concerns the mobilisation of human resources and the incorporation of all the elements within a whole, including the policy-makers, the public and private sectors, the students, the teachers and the parents (25). The major obstacles reported by European countries include the need to reinforce cooperation among the different sectors, including the education, health, technical and support sectors, as well as having access to sufficient funds (26). The results of the only study conducted in Iran in East Azerbaijan revealed most barriers and challenge to be related to inter-sectoral cooperation, policies and rules, infrastructures and capacities, human resources and social cooperation (27).

However, there is insufficient data regarding the need of students to develop a health promoting school programme in Iran. Therefore, the present study was conducted to investigate the current approach of schools to health and to clarify the health needs of adolescents as well as the challenges encountered through the views expressed by the students themselves and their teachers in Zahedan, Iran. Furthermore, for girls, adolescence is the foundation of their future; since it guides the next stages throughout their lives and directly affects their future family and children. Therefore, investing in female adolescent health is essential because of their two-fold effect on the health of the community and that of impending generations. Consequently, the present study focused on female students.

## Materials/Subjects and Methods

This paper is based on the findings of a small-scale explorative qualitative research project originally intended to provide input into the design and development of a plan on the health promotion of female student in South-East, Iran. Two schools were selected from among 18 governmental girls high school in Zahedan (Iran) which participated in healthy school plan based on cooperation statement reported by school authorities.

This purely exploratory research was carried out for four weeks (Sep–Oct 2015) on a sample of 30 students and 15 teachers.

The inclusion criteria for the required participants were to be grade ten students and full-time teachers. The students were selected through random sampling whereas the teachers were selected by purposive sampling method. An important element during the initial planning stages of this research was relevant types of questions used in the face to face semi-structured interviews with the students as well as four focus group discussions conducted on the teachers in groups of six to eight. Open-ended questions seemed to be most suitable for the present study. Unknown questions were added if necessary as the interview process progressed. Indeed, interviews’ performance stopped when no new needs for new question emerged.

Interviews with the students continued until saturation of data. Correspondingly, all participants were asked to discuss with respect to the guide questions stated by the researcher. Then, topics were collected by the navigator in the last 15 min. The interviews and focus group discussion lasted for approximately 45–60 min and 60–90 min, respectively.

Additionally, participants’ verbal communications were recorded under the condition that the files would be destroyed after analysis. All interviews and focus group with participants were carried out/coordinated by main investigators (Fariba S-S and Fatemeh R). Moreover, participants were offered a choice of locations and times to ensure the confidentiality of the interview process.

The ethical considerations observed in the study included presenting an official introduction letter, explaining the study objectives, emphasising the role of the researcher and participants, obtaining informed consent and ensuring the confidentiality of the data. Additionally, they were given the option of taking notes instead. Following the interviews, participants were asked if they had any additional questions. To ensure conformability and credibility, several methods were used, including bracketing, prolonged engagement, concurrent analysis and peer review.

All analyses were performed using MAXqda11. The constant comparative analysis of the students’ and the teachers’ responses was carried out in three coding stages, including open, axial and selective coding. Overall, two sets of data were analysed; the first set of data consisted of interviews with the students, whereas the focus group method with the teachers was utilised to collect the second form of data.

The analyses were initiated by reading texts frequently to find a general sense of interest. Then the texts were read word for word to extract keywords for final analysis.

Three readers used open coding in the multiple evaluating techniques to identify the major idea and needs to be revealed in the participant’s words, phrases, statements, and examples. After that the readers integrated defined needs were obtained from the interviews and group discussions. Initial needs were thus classified, similar needs were integrated, and classes and sub classes eventually formed.

## Results

The present exploratory qualitative study was conducted on 30 female students in the tenth grade and 15 teachers in the South East of Iran. The students were in the range of 14–17 years old and majority had a birth order of 1–3. In terms of family relationships, a minority of them mentioned that they always speak with their parents and are involved in the decision making process of their family. The characteristics of the participating students showed that majority of the students’ fathers, and about half of their mothers were employed and one third of the students had no sisters (Table 1). From the responses of the students and teachers, a total of nine needs were identified.

### Need 1

Informing the students about the aims of the schools’ health project.

Nearly all the students declared they had no information on the school health project with regards to empowering students, except for one, who revealed, she had heard a little about it on the radio. Regarding the health measures taken during the academic year, the students only mentioned height and weight measurements and eye examinations performed by the school health officer, and psychiatric health tests given by the school counselors. Study subjects additionally mentioned who have got some information about HIV, the flu and waterborne diseases from out-of-school experts. Furthermore, the distribution of iron supplements in the school reported by students as well. Teachers also believed that the plan was not successful in school. However, it might be successful if there would be effective intervention.

### Need 2

Education and training on all dimensions of health with an emphasis on mental health.

As for the question ‘how useful is the health-promoting schools project?’ The majority of students believed that this project could be very beneficial. For example, one of the students (Student 25) said: “I am interested in my own health, but those who talk on this issue keep saying that it should be like this and not like that, and I cannot be bothered with all that. It would be fine if the project was implemented in an attractive way with a lot of variety so that it wouldn’t be so boring.”

The summary of the views expressed by the teachers showed a unanimous agreement for the implementation of the health project in the schools, only if all dimensions of health were simultaneously and fully implemented. In comparison to students, the teachers believed that the psychological dimension of health should have the highest priority in the performing of interventions.

### Need 3

Use of experts in various fields of education from other organisations.

Almost all students and teachers unanimously suggested that it was better to have experts in various fields present in the school instead of having their counselors or teachers present in the subjects. The reason for their mentioned preference was that experts are more experienced and have expertise in a particular area. Additionally, the education provided by experts is necessarily more than and more extensive as they are able to present various effective solutions. For instance, one of student stated (Student 3), “Since their education is focused on one specific area, experts have more experience and are in a better position to guide and help us. It is best to learn things from the main source rather than from someone who has had to be trained first and has now decided to teach us. I think young experts understand us foremost and can be more useful.” In addition, teachers noted that “students interacted with strangers better than them, as they are more attracted to the strangers than the teachers.” A minority among the students argued for the superiority of peer education due to the slighter communication and education by the teachers, due to their easier availability. In contrast, teachers believed on peer training for its greater effectiveness.

### Need 4

Employing capable and trusted counselors in schools.

The students mentioned that skilled counselors were more experienced and are specialised in providing information. Therefore, they are capable of providing more effective strategies in schools. However, the biggest reason for the students not wanting to see counselors was their lack of trust in them, the reason is that: “as soon as we talk to a counselor, the school officials and our parents get to know about our problems, too. So they cannot be trusted to keep a secret” (Student 20), and “I like to finish whatever I have started by myself and work out my problems myself” (Student 24).

Furthermore, all the teachers believed that the presence of experts and experienced counselors adept in the debate was highly essential for schools since these new people would be more attractive for the students and would be listened to with more enthusiasm, but the students preferred consultants from outside the school because they are more reliable.

### Need 5

Use of a variety of educational methods.

The students asserted that practical training and group discussions are more effective strategies, as it allows them to discuss and solve whatever concerns or questions come to mind on the spot. The use of educational audio-visual media was the students’ second choice, due to their greater efficacy and longer-lasting impact on their mind. They added that if the presented media were in the documentary form with people sharing their past experiences, the students would be more tangibly involved in the subject. Written educational materials (books, booklets, pamphlets, etc.) were the students’ third choice, as they believed these materials could be used more frequently and could be better understood. A number of the students, however, believed that studying written materials was boring.

In addition, nearly all the students expressed that, they had access to the Internet via their cell phones and believed that the student health website would be very effective if the materials were up-to-date and interesting. Furthermore, counselors could respond to the students’ questions online. Only a small minority said that they had no access to the Internet or had no interest in surfing the web.

The teacher added that group discussions and personal teaching are more effective. They recommended the screening of educational video clips and short films across schools. Furthermore, educators with a different view have stated that since parental cooperation was poor, a series of teaching materials should be given to the students to take home to their parents. Teachers then suggested that the school should confidentially invite the parents of troubled students for psychological counseling, in order to enhance their problem-solving skills and solve the actual problem their children are having.

### Need 6

Using reward systems for encouraging the student’s cooperation with group activities.

Furthermore, almost all the students asserted that they preferred individual activities at home over group activities within the school, because they found singular activities to be easier as they only had to deal with their own opinions, and as they did not have to consider the collective views. In addition, if a mistake is made, they could fix it without worrying about being ridiculed by their peers. They also believed that they could concentrate better on individual activities and therefore, obtain the best performance. Moreover, they said that their privacy was more respected at home when no one else would watch over their activities.

Similarly, the teachers also argued that nowadays, most students are busy with their cell phones and tablets or might be in touch with each other online. For instance, one of the teachers stated the following: “I am a physical education coach. Most kids bring letters prohibiting them from exercise. They are not interested in exercising at all and only like to chit-chat with each other.” They suggested that the reward system should be used to increase the participation of students.

### Need 7

Teaching communication and the ability to establish good relationships with the parents and strategies for resolving family conflict.

One-sixth of the students said that they discussed their problems with their parents (father or mother). In contrast, a number of the students expressed concerns about talking with their parents and almost unanimous in stating that their most urgent issue of the present moment was to establish a good communication with their parents. Some of the statements made by the students are provided in the following section as examples.

“They have not at any time talked to me and not for a moment asked me about my problems. Only when I do something wrong will they come and scold me, instead of solving my problem” (Student 11). “I have problems with my parents. I hate them. I have done something wrong, and now they keep bugging me about it” (Student 18). “My father picks on me for the slightest of things. It has gotten so bad they won’t even let me study. I have so many problems that I like to come to the school straight on Fridays. I sometimes envy other kids who like to go back home as quickly as possible” (Student 19). “They have never talked to me about my problems” (Student 30). “My parents discriminate between me and my sisters, and this is a big concern of mine. My father says that a girl is like a pearl in a shell, and it is the father’s job to protect her. However, he doesn’t trust me. He says that trust is like a piece of paper that will never be like it was before when it’s crumpled. My biggest concern is that he doesn’t trust me. He compares me with others, and I don’t like being compared” (Student 29).

“I am having a mental breakdown. Whenever I decide to start doing something they do not encourage me, instead, they all say that I will fail. For example, when I wanted to study medicine, they all mocked me and told me that I will not get admitted” (Student 1). “I just do not get along with my mother. I hate her. She tells me what to wear, what makeup to put on. She forced me to wear the veil. She wants me to do things that will make me get ridiculed by everyone” (Student 7). “They do not let me wear things I like to parties. They do not let me go to my friend’s house. They are very suspicious” (Student 6).

“They bring up the one mistake I once made and just won’t let go. I cannot talk to them” (Student 4). “I am worried about how they are going to treat me when I get home. My parents don’t understand me. I like to be an actress, but they keep telling me I should study, and only in the summer do I get to do what I really like to be doing” (Student 12). “They’re constantly fighting in our house, and so my grades dropped” (Student 10). “I’m not close with my family, and I don’t get along well with them. I am by myself most of the time. They are so busy, and are usually out” (Student 16). “They make fun of me all the time, and tell me to be quiet. When I ask for something, they tell me that I can do whatever I want to do when I get older and get a job” (Student 18). “My father will not even take us out on Fridays. I wonder why he is so different. Sometimes he picks on us and argues. My family treats my father so negatively. They always provoke him. When I suggested that he should go for counseling, he said that he was not crazy” (Student 2).

In this regard, one teacher asserted, “The students don’t have many physical problems, or at least; they’re doing fine, but most of them have family problems.” The next teacher said, “Most of the students have had upbringing problems. I think many parents don’t have any parental competence at all. All the problems that these kids have to deal with are related to their family.” Another teacher believed that “The problem rests with the parents. They don’t come into the school very often, and their collaboration in the parents-teachers meetings is minimal. The school officials have to pressure them into attending the meetings.” Longer teacher viewed, “It is as if the kids with parents don’t really have parents. Most parents are emotionally divorced.” One teacher declared, “The parents justify their children. They haven’t been trained for parenthood. I had a student whose parents had gone away on a month’s holiday and had left the girl with her brother… she suffered academically.”

### Need 8

Educational high-risk behaviours and strategies for handling them for parents and students.

According to the teachers, the major problems of the students included behavioral and moral problems, drug abuse, and relationships with the opposite sex, aggression and violence, loss of motivation and the sense of responsibility, sedentary lifestyle, and poor guardianship.

One of the teachers stated that, “Today, the mass media; the Internet and satellite TV have a huge influence on the behavior of children and most kids develop behavioral problems.” Another teacher said that, “The number of objectionable files on their tablets is very alarming.” Another teacher also stated that, “It is as if not having a friend of the opposite sex makes students to feel stigmatised these days, they think they are old-fashioned if they do not have one.” Another teacher explained that “When one of them finds a boyfriend, she becomes an intermediary between her friends and friends of her boyfriend. “Furthermore, most students play the victim as if they are innocent … and their parents back them up.”

One of the teachers believed that “It has to do with having one or two children only. Sometimes you’re going to have a lot of problems if you have simply addressed them in the familiar form.” Other teachers added, “The parents put up their defenses as soon as we tell them about their kid’s problems.” Additionally, they viewed that, “Parents make so many obvious mistakes in front of their kids nowadays that give them an excuse, and they can no longer control their kids.” One of the teachers said that “It is the parents’ relationships outside the home that are to blame for the kids’ problems.” The next teacher opined that, “A lot of mothers are addicted nowadays. Women with the problem of addiction cannot raise their children properly, and their kids do not listen to them. Parents have generally lost their position of respect within the family.”

Lastly, teachers provided the recommendations after summarising the points discussed for improving the current status including, holding educational workshops for the students and parents, psychological-sexual training about relationships with the opposite sex and resolving the emotional dependence of these relations for students, training on how to manage puberty problems for parents, screening documentaries about the life of troubled people as a warning to the students and inviting successful people to become the student’s role-models.

On the other hand, the students said that they wanted to learn more about friendly relationships with friends, the public and the parents, skills for making friends, talking skills, the ability to gain others trust and the ability to take decisions. Additionally, to increase their ability to say no against risky behaviours, teaching about the protection of privacy on public networks and the cyberspace and teaching to control high-risk behaviours.

### Need 9

Reforming wrong attitudes and indigenous sub-culture.

Teachers believed that cultural issues also cause problems for example, “We have a student with thalassemia whose father forbids from receiving blood transfusion” or “The Iron supplementation plan for teenage girls … no training or information was provided for the parents. This failed as a result of the parents’ wrong attitudes. The students threw the pills into the trash straight away, and when asked why, they disclosed that their parents had told them not to use the pills because it may later result to infertility.”

According to the teacher’s views, the major problems of the students included behavioural and moral problems, relationships with the opposite sex, aggression and violence, loss of motivation and the sense of responsibility, cultural and upbringing problems, malnutrition, family’s financial hardships, sedentary lifestyle and poor guardianship.

The focus group discussions with teachers along with interviews with students produced the eight main themes including the project and its implementation, financial resources, project management, human workforce, educational facilities, social and demographic factors, educational resources and educational priorities as well as 22 sub-themes (Table 2) to be considered in developing health promoting school programme.

## Discussion

The main contribution of the present study is that, so far no study has been done to search for needs of the student in order to design intervention and health promotion programme based on the views among the students and teachers. This qualitative study is a narrative interview with female students as well as discussion with their teachers. The project and its implementation, financial resources, project management, human workforce, educational facilities, social and demographic factors, educational resources and educational priorities were the main themes for better performance of health promoting programme obtained from students and their teachers ideas. These issued followed by some sub-themes stepwise and continuous training, use of experts in various fields, timing fits, participation of parents and provide sufficient funds; come from discussions and interviews.

Overall, both teachers and students believed that, this project could be successful only when all the components of health promotion were implemented in schools under one comprehensive project consisting of health education, physical activity, nutrition, high-risk behaviours, health examinations and psychological counseling, the promotion of teachers’ health, encouraging parental cooperation, and creating a physically healthy environment. The findings of the current study are in line with the research which reported that the school’s role was quite clear as an environment where most of the individual’s waking hours are spent during a certain period to her life, where friendships are formed and socialisation begins and which has many other functions as well.

Experts believe that schools can have a valuable role in evaluating the healthcare that the students do not receive elsewhere (28). However, according to the students and teacher’s views, the health services provided at schools were inadequate and sub-standard. The discontent with the school is a phenomenon also reported in wealthier Western countries (29). A study by Tavafian and Molaei showed unfavourable health-promoting behaviours, and a consistently sedentary lifestyle in the students examined (30).

These findings corroborate a multiethnic styrene study which concluded that the health promoting programmes of schools can be well-known if they are holistic and focused on certain groups, and if they have a clear evaluation criteria (31). Likewise, the teachers suggested that schools’ health promotion projects could be successful with the allocation of sufficient funds, comprehensive planning, effective management and ongoing implementation; they also believed that getting the assistance of the school heads, the cooperation with the teachers, and the participation of the parents can play a major role in facilitating the stages of student training.

The majority of the teachers and the students declared that the main problems were in mental areas and expressed the need for psychological counseling with experienced consultants outside the organisation. In line with results from other studies, it should be noted that mental health programmes with a focus on schools (i.e. the employees, teachers and parents) and the design of curriculum compatible with adolescents’ needs should be the top priority to the government for policy-making (32). The provision and development of schools’ psychological health services and programmes play a vital role in identifying the unique needs of students who come into the school with numerous psychological problems every day (33).

A study entitled “the students’ need for counseling to gain real-life experiences” argued that students need to gain real-life experiences through their counselors, and that they have no interest in being preached to; however, preaching may benefit them. The study further argued that students need tangible examples of difficult actual life situations or perhaps need counselors who can help them understand and face actual life situations (34). Gilman et al. believe that students need guidance, encouragement and practice in the collection, storing and analysis of advice and necessity to be able to debate them, and if these needs are not systematically identified, reasonable satisfaction will not be achieved (35). Their coping responses indicate that the students revealed their need for counselors who can provide them with practical and effective strategies. They also said that they preferred out-of-school counselors since they did not trust the school counselors.

The majority of respondents in the present study voiced worries that the main problem among the students was concerned about their relationships with their parents and family. Other researchers have pointed that the students’ link with their school plays a mediator role in their behavioral problems as well as their relationship with their parents (36). Nevertheless, if the students are unable to develop their relationship with their family, they may not be capable to benefit much from the link in the school either, and however, they might count on the teachers and the other students, and if they find them caring, they will not be skillful to benefit from these sources in achieving success or avoiding high-risk behaviours (37).

The fact that students are most influenced by their parents and family members in the domain of social development and education, acquiring social skills through the family can be better accomplished with proper family-oriented planning and the highest level of investment in training the families. It is, therefore, essential in the education system, which is in direct contact with the families, to teach the required social skills to the families.

The teachers claimed wrong parental attitudes to be another challenge which they had to deal with. It is, therefore, important to give parents awareness about their vital role in conveying correct and adequate information to their children and to essentially change their attitudes. This information should be provided at the right time and in accordance with the needs of the audience and the society’s dominant culture.

Technological advances have created numerous positive and negative complications and consequences for the individual and the society. Castles believed that modern technologies create new identities for individuals and communities through facilitating the emergence of a network community. These recent identities affect people’s interactions with each other and also have the capacity to challenge social structures and institutions. Students use cell phones for communication and are undoubtedly affected by this issue. The results of previous studies suggest that cell phones’ attraction for adolescents creates complications such as the loss of self-esteem and social confidence, isolation, sense of responsibility, cooperation and respect for values (38).

Moreover, a new form of privacy has emerged among adolescents, especially among students, and cell phones have become a popular means of providing this privacy. This privacy can have numerous effects on adolescents and young adults and can aid the formation of inappropriate relationships with peers, especially of the opposite sex, which is eventually considered a threat to the individual’s psychological health (39). The teachers who participated in the present study also expressed concerns about the students’ use of cell phones, virtual social networks, and the Internet and their effects on the students’ morals and behaviours.

Undoubtedly, cell phones are the most widely used and the most popular technology in today’s society. Offering such facilities as sending text messages, emails and photos and connecting to others through the Internet and via Bluetooth, this communication device has become an inseparable part of life in the modern world. Any new phenomenon that emerges in the society has positive and negative functions, which should both be considered during the examination of a phenomenon. Despite the threats it poses and the limitations it imposes, the cyberspace has a lot of potentials as well. Policy-makers should first identify adolescents’ needs and then proceed to properly manage this space (accessed through cell phones) and direct it toward producing appropriate and safe content in formats attractive to adolescents.

Traditional educational methods have the advantage of presenting a large volume of subjects in a limited time span, but they are much less effective compared to modern methods in nurturing thinking, creating motivation and changing attitudes and they tend to waste resources. The use of alternative methods of education thus seems essential, as they provide longer-lasting and more efficient learning and create greater interest in learning (40). Previous studies have shown that when the students’ process of learning is active, their motivation for learning increases, and they become better learners. The use of multimedia technologies, including computers, appears to be impressive in improving the quality of education and the interest in it (41). Both students and teachers who participated in this study viewed that modern media such as video clips, audio files, films, group discussions and practical activities can be more effective in education and learning. The majority of participants also liked the design on the web page on health.

The main teaching needs of participating in this study included acquiring proper models of family relationship, resolution strategies in family conflict and methods of controlling high-risk behaviours. The finding from this study provided more support for Parvizi et al. study, in that, they reported that adolescents considered living in a healthy society as the foremost indicator of health and emphasised the effect of cultural-educational facilities on their health. They also listed their most essential needs on the order of their significance, including receiving sex education, having a healthful family, increasing the parents’ knowledge and skills for handling adolescents’ needs, addressing psychological health issues and having a healthy diet. In contrast, unhealthy relationships were cited as a major threat to health, and the participating adolescents emphasised the positive and negative effects of forming relationships with the opposite sex (42).

Overall, there is some evidence that interventional programme based on school needs and in line with usual aims of school along with teacher cooperation in training and development as well as supporting programmes, could play an essential role in the success of health promoting schools (43). A study also reported that five factors enabling the programme included the framework of programme, support and collaboration of responsible managers and school staff, policy makers, health care organisations and stakeholders in health, establishment of multidisciplinary approaches, collaborative programmes; networking and professional relationships, and continuing education in their schools (44).

Additionally the data suggest that four theories must be considered in designing interventional programmes including; preparation programmes for implementation of the consultation before the actions, defined programme in each school, embedded application in usual and routines schools activities, health implementation and compliance with the programme (45). Similarly a study reported eight necessary components to implement health promoting schools approach such as planning, preparation programmes, the political and organisational support, students’ participation, networking, partnerships and sustainability programmes (46). The results of the present study were similar to previous studies and nine factors were identified to be effective in the implementation of intervention within healthy school programmes.

## Conclusion

Exact noteworthy findings in line with the needs of student include attracts more participation of parents with school heads and teachers, using experts from different fields, engaging teaching approaches, using powerful and experienced psychological counselors, holding workshops for parents and students, particularly about family relationship, risky behavior and specific problems in adolescence duration and developing a comprehensive educational content with regard to cultural issues and attitudes.

The strengths of the study are that the design intervention is based on the real needs of students. In addition, the study has been conducted for the first time in the region in that, its finding could play a valuable role in health promoting school policy. Nevertheless, certain issues are poorly understood. Thus we recommend further studies in all age and gender groups.

One limitation of the current study is the nature of qualitative studies in which the results of the present study cannot be highly generalised to other societies; however, the strategies adopted for ensuring the acceptability and objectivity of the data yielded findings with a good validity and reliability.

## Figures and Tables

**Table 1:** Demographic characteristics of students

Variables	Frequency (%)
Father’s job	
Employment	26 (86.6)
Unemployment	2 (6.7)
Dead	2 (6.7)
Mother’s job	
Employment	13 (43.3)
Housewife	17 (56.7)
Father’s Education	
Primary	3 (10.0)
Secondary	7 (23.3)
High school	6 (20.0)
University	14 (46.7)
Mother’s Education	
Primary	6 (19.9)
Secondary	8 (26.7)
High school	8 (26.7)
University	8 (26.7)
Number of sister	
No	9 (30.0)
One	8 (26.7)
Two	6 (20.0)
Three	7 (23.3)
Number of brother	
No	14 (46.7)
One	10 (33.3)
Two	4 (13.3)
Three	2 (6.7)

**Table 2:** The main themes and sub-themes extracted

• Funds - allocate appropriate funding - to provide sufficient funds	• Programmes and Performances - education and training in all areas of health - stepwise and continuous training - consistency in the application and planning
• Project Management - the commitment of members - the timing fits - consider operational issues	• Human Resources - use of experts in various fields - utilisation of scientific capacity, experience and performance in the area of operation
• Training Facilities - assist school administrators in interventions - the cooperation of teachers in training programs - participation of parents in the program	• Social and Demographic Factors - taking into account local subcultures - corrected wrong attitudes
• Educational Resources - providing a diverse and attractive content - Presentation of a new field of health - use a variety of educational methods	• Educational Priorities - family communication patterns - strategies to resolve family conflicts - risk behaviour - mental health
